# The Effect of American Society of Anesthesiologists Score and Operative Time on Surgical Site Infection Rates in Major Abdominal Surgeries

**DOI:** 10.7759/cureus.55138

**Published:** 2024-02-28

**Authors:** Rayees Ahmad Bhat, Natasha Varghese Isaac, Jeffin Joy, Deepika Chandran, Kevin Joseph Jacob, Samantha Lobo

**Affiliations:** 1 Department of Surgery, Hamdard Institute of Medical Sciences and Research, New Delhi, IND; 2 Department of Medicine, St. John's Medical College Hospital, Rajiv Gandhi University of Health Sciences, Bangalore, IND; 3 Department of General Surgery, Jubilee Memorial Hospital, Trivandrum, IND; 4 Department of Vascular Surgery, Starcare Hospital, Calicut, IND; 5 Department of Surgery and Trauma, Christian Medical College, Vellore, IND; 6 Department of Surgery, Ukrainian Medical and Stomatological Academy, Poltava, UKR

**Keywords:** risk of surgical site infections, asa grade, surgical site infections, mean duration of surgery, asa score

## Abstract

Background: The objective of this study was to evaluate the influence of two crucial variables, the American Society of Anesthesiologists (ASA) score and operative time, on the occurrence of surgical site infections (SSIs) in the context of major abdominal surgical procedures.

Methodology: A cross-sectional research study involved patients undergoing various gastrointestinal surgical procedures. Surgical details, procedure duration, and ASA score were meticulously documented. Patients were observed for surgical site infections (SSIs) during their inpatient stay until discharge. Following their discharge, patients were monitored in the outpatient department for a minimum of 30 days post-surgery, and those who underwent mesh procedures were observed for one year.

Results: In the overall study population, surgical site infections were identified in 42 cases, constituting 6.7%. There was a significant association between ASA grade and the incidence of surgical site infections (p=0.001), indicating a higher prevalence of infections in cases with elevated ASA grades. Furthermore, a statistically significant association exists between the average duration of surgery and the occurrence of surgical site infections (p=0.001). The mean surgery duration for cases with infections is reported as 206.33 min, with a standard deviation of 103.73, while for cases without infections, the mean duration is 99.72 min, with a standard deviation of 79.71. In the multivariate analysis, it was found that an ASA score of 3 or higher and operative time exceeding 90 min were identified as independent factors for predicting the likelihood of surgical site infections.

Conclusion: The significant associations identified between the American Society of Anesthesiologists (ASA) grade, average surgery duration, and SSIs underscore the importance of comprehensive preoperative assessment and procedural management in infection prevention.

## Introduction

Surgical site infections (SSIs) still remain an important concern in major abdominal surgeries. SSIs can lead to extended hospital stays as patients may require additional medical attention, wound care, and monitoring for signs of infection. This not only impacts the patient's recovery but also increases healthcare costs [1].

The financial burden associated with treating SSIs is substantial. It includes the costs of extended hospitalization, additional medications, diagnostic tests, and potential surgical interventions to address complications [2]. SSIs contribute to patient morbidity by causing pain, discomfort, and potential complications. In more severe instances, SSIs have the potential to progress to systemic infections, presenting a more substantial risk to the overall health of the patient [3]. SSIs can compromise the success of the surgical procedure itself. Infections may lead to wound dehiscence, abscess formation, and the need for secondary surgeries, which negatively impact the overall surgical outcome [4].

As healthcare providers strive for enhanced postoperative outcomes, understanding the multifactorial nature of SSIs becomes imperative. This study aimed to examine the influence of two crucial variables, the American Society of Anesthesiologists (ASA) score and operative time, on the occurrence of surgical site infections (SSIs) in the context of major abdominal surgical procedures.

The ASA score is utilized as an indicator of a patient's preoperative health condition and the presence of comorbidities [5]. It is crucial to explore its impact on surgical site infections (SSIs) in major abdominal surgeries, given that elevated ASA scores are linked to an increased likelihood of complications, including infections [6]. Operative time is a key variable due to its association with the duration of exposure to the surgical environment. Prolonged operative times can increase the risk of SSIs due to extended exposure to potential pathogens, compromised tissue perfusion, and the need for more complex procedures [7].

The ASA score, a widely accepted metric for preoperative patient assessment, reflects the overall health status and comorbidities of individuals. While previous research has linked higher ASA scores with increased perioperative risks, the specific relationship between ASA score and the incidence of SSIs in major abdominal surgeries remains an area of active investigation. Similarly, operative time, a variable influenced by various surgical factors, has been implicated as a potential contributor to postoperative complications, including SSIs. This study aimed to fill existing knowledge gaps by rigorously examining the interplay between ASA scores, operative time, and the likelihood of SSIs. Through a comprehensive analysis of patient data from major abdominal surgeries, we seek to elucidate whether higher ASA scores and prolonged operative times elevate the risk of SSIs. Ultimately, the findings of this research may guide clinicians in refining preoperative risk stratification and optimizing surgical strategies to mitigate the incidence of SSIs, fostering improved patient outcomes in major abdominal surgeries.

## Materials and methods

A cross-sectional research investigation was carried out at the Department of Gastrointestinal and Bariatric Surgery in BLK Super Specialty Hospital, New Delhi. Data collection spanned one year, from January 2017 to January 2018, involving patients who underwent various gastrointestinal surgical procedures, such as esophagectomies, gastric resections, pancreaticobiliary procedures, enteric and colorectal resections, as well as hernia repairs, using either conventional or laparoscopic methods. The study encompassed both elective and emergency surgeries. The study was approved by the Institutional Ethical Committee of BLK Super Specialty Hospital, New Delhi, India (#129/2016).

During the preoperative phase, comprehensive patient data, including demographic information and detailed medical history, were collected, along with preoperative examinations and relevant investigations. All patients received preoperative antibiotics. Surgical details, including whether the procedure was elective or emergency, laparoscopic or open, duration of the procedure, intraoperative findings, and contamination during surgery (wound class), were meticulously documented.

Patients were observed for surgical site infections (SSIs) during their inpatient stays until discharge. Following their discharge, patients underwent postoperative follow-up in the outpatient department for a minimum of 30 days, and those who underwent mesh procedures were monitored for a duration of one year. The classification of surgical site infections (SSIs) was based on the Centers for Disease Control and Prevention (CDC) definition, differentiating between superficial, deep, and deep, and organ or space infections [8].

Data collection involved a semi-structured form, and data entry was performed using Excel. The data underwent outlier screening, and analysis was conducted using SPSS version 25.0 (Armonk, NY: IBM Corp.). Categorical variables were presented as proportions and percentages, while continuous variables were expressed as mean and standard deviation. Measures of association were determined using the odds ratio and mean comparisons utilizing the independent t-test. A p-value less than 0.05 was considered statistically significant.

## Results

Table 1 describes the distribution of surgical site infections (SSIs) based on various categorical variables used in the study. In the studied population, surgical site infections were observed in 42 (6.7%) cases, while they were not present in 584 (93.3%) cases. The study reported a significant association between surgical site infections with gender (0.001), wound class (0.001), surgery indication (0.001), surgery type (0.001), and hypoproteinemia (0.03). No statistically significant association is observed between surgical site infections and comorbidity (0.89).

**Table 1:** Baseline demographics and clinical characteristics of the study population. P-value <0.05 was considered statistically significant.

Variables		Surgical site infections, n (%)		p-Value
		Present, n= 42	Absent, n =584	
Gender	Female	13 (31.0)	306 (52.4)	0.001
	Male	29 (69.0)	278 (47.6)	
Wound class	Category 1	2 (4.8)	84 (14.4)	0.001
	Category 2	19 (45.2)	451 (77.2)	
	Category 3	4 (9.5)	15 (2.6)	
	Category 4	17 (40.5)	34 (5.8)	
Surgery indication	Elective	18 (42.9)	527 (90.2)	0.001
	Emergency	24 (57.1)	57 (9.8)	
Surgery type	Laparoscopic surgery (L)	7 (16.7)	467 (80.0)	0.001
	Open surgery (O)	24 (57.1)	87 (14.9)	
	Laparoscopic converted to open (LO)	11 (26.2)	30 (5.1)	
Comorbidity	Absent	28 (66.7)	391 (67.0)	0.89
	Present	14 (33.3)	193 (33.0)	
Hypoproteinemia	Present	13 (31.0)	105 (18.0)	0.03
	Absent	29 (69.0)	479 (82.0)	
Hypoalbuminemia	Present	7 (16.7)	33 (5.7)	0.001
	Absent	35 (83.3)	551 (94.3)	

Table 2 describes surgical site infections based on their types, indicating that the majority of infections were superficial, 21 cases (50.0%), followed by organ or space infections, 13 cases (31.0%), and deep infections, eight cases (19.0%).

**Table 2:** Incidence of surgical site infection.

Type of surgical site infections	n (%)
Deep	8 (19.0)
Organ or space	13 (31.0)
Superficial	21 (50.0)
Total	42 (100.0)

Table 3 demonstrates the relationship between the American Society of Anesthesiologists (ASA) grade, average surgery duration, and the incidence of surgical site infections.

**Table 3:** Bivariate analysis of ASA grade and mean duration of surgery with surgical site infections. P-value <0.05 was considered statistically significant. ASA: American Society of Anesthesiologists

Variables		Surgical site infections, n (%)		p-Value
		Present	Absent	
ASA grade	Grade 1	9 (21.4)	231 (39.6)	0.001
	Grade 2	13 (31.0)	296 (50.7)	
	Grade 3	13 (31.0)	55 (9.4)	
	Grade 4	7 (16.7)	2 (0.3)	
Mean duration of surgery (mins) - mean/SD		206.33 (103.73)	99.72 (79.71)	0.001

Table 4 highlights a notable correlation between ASA grade and the occurrence of surgical site infections (p=0.001), indicating a higher prevalence of infections in cases with elevated ASA grades.

**Table 4:** Multivariate analysis of risk for surgical site infections. P-value <0.05 was considered statistically significant.

S. no.	Risk factor	Coefficient	SE	p-Value	OR (95% CI)
1.	ASA score (≥3)	0.759	0.249	0.002	3.13(2.31-4.48)
2.	Operative time (≥90 min)	0.918	0.612	0.134	2.50 (0.75-8.31)

Furthermore, a statistically significant association exists between the average duration of surgery and the occurrence of surgical site infections (p=0.001). The mean surgery duration for cases with infections is reported as 206.33 min, with a standard deviation of 103.73, while for cases without infections, the mean duration is 99.72 min, with a standard deviation of 79.71. In the multivariate analysis, it was found that an ASA score of 3 or higher and operative time exceeding 90 min were identified as independent factors for predicting the likelihood of surgical site infections.

## Discussion

The results of the study offer valuable insights into the link between the American Society of Anesthesiologists (ASA) grade, average surgery duration, and the occurrence of surgical site infections (SSIs) in gastrointestinal surgeries. The study findings emphasize the importance of both preoperative health status and procedural factors in influencing postoperative complications.

The statistically significant association between ASA grade and the occurrence of SSIs is consistent with existing literature. Higher ASA grades, indicative of a patient's overall health status and comorbidities, have been recognized as predictors of surgical complications, including infections. The results of the present study demonstrate an incremental increase in the prevalence of SSIs with higher ASA grades, suggesting a correlation between preoperative health conditions and postoperative outcomes. Clinicians should, therefore, consider ASA grade as a valuable tool for risk stratification and preoperative decision-making. A reported ASA score of three or higher has been linked to a significant rise in the occurrence of surgical site infections compared to those with a score below three [9]. Individuals with elevated ASA scores may have compromised immune systems as a result of underlying health conditions. A diminished immune system can pose increased difficulty for the body in resisting infections, including those occurring at the surgical site [10]. Likewise, in a recent study conducted by Mulita et al. on patients who had undergone colorectal surgeries, the incidence of sepsis showed a significant association with ASA score >2 [11]. In another study done by Panos et al., post colorectal resection procedure SSIs were more common in patients with ASA scores >2 [12]. Higher ASA scores are often associated with chronic medical conditions, such as diabetes, cardiovascular disease, or respiratory disorders. These conditions can impact the body's ability to heal and increase the risk of postoperative complications, including infections [13].

Patients with elevated ASA scores may be undergoing more complex surgical procedures, potentially causing prolonged operative durations and an increased susceptibility to contamination. The complexity of the procedure itself can contribute to the heightened risk of surgical site infections (SSIs). The observed association between the average surgery duration and surgical site infections (SSIs) aligns with the concept that extended operative times may play a role in elevating the likelihood of postoperative complications. The statistically significant association highlights the importance of optimizing surgical techniques and strategies to reduce procedural duration, minimizing the potential for infections.

Several multicenter studies have consistently identified a statistically significant correlation with prolonged operative time, except for a single study [14]. Notably, around 95% of studies with a sample size exceeding 1,000 patients reported a statistically significant association. Many studies indicated that operative time remains as one of the few independent predictors of surgical site infections (SSIs) [15-20]. Some studies even emphasized that an extended operative time was identified as the most crucial, or sole, risk factor for SSIs [21,22].

The duration of surgery serves as an independent risk factor for surgical site infections (SSIs) and is potentially subject to modification, unlike certain patient-related risk factors such as the presence of diabetes mellitus. Various factors, including preoperative planning, surgeon expertise, surgeon fatigue, experience of operating room staff, and the availability of equipment, can influence the operative time. Although the precise mechanisms through which prolonged operative time contributes to an elevated incidence of surgical site infections (SSIs) are not completely comprehended, several studies propose plausible explanations.

Prolonged operative time exposes patients' open incisions to the environment for an extended duration, thereby heightening the risk of bacterial contamination. Additionally, longer operative time predisposes incisions to tissue desiccation, which may also escalate the likelihood of contamination [23,24]. Surgeons and healthcare providers should consider time management in the operating room as a modifiable factor in the broader context of infection prevention protocols.

Incorporating ASA grade into the preoperative assessment allows for effective risk stratification. Patients with higher ASA grades may benefit from additional preventive measures and closer postoperative monitoring. Emphasizing the correlation between mean duration of surgery and SSIs underscores the importance of procedural efficiency. Implementing strategies to streamline surgical procedures may contribute to a reduction in postoperative complications. The identified associations provide a basis for the development of targeted interventions. Tailoring preventive measures to high-risk groups, particularly those with elevated ASA grades or undergoing longer surgeries, may enhance overall patient outcomes.

While the results offer valuable insights, certain limitations should be acknowledged. The single-center design of the study may introduce biases, and the generalizability of findings to diverse populations should be approached with caution. Future research endeavors could involve multicenter studies and explore additional variables that may contribute to SSIs, such as intraoperative techniques and postoperative care protocols.

## Conclusions

In conclusion, the present study highlights the intricate relationship between preoperative health status, mean duration of surgery, and the incidence of surgical site infections (SSIs) in gastrointestinal surgeries. The significant associations identified between the American Society of Anesthesiologists (ASA) grade, average surgery duration, and SSIs underscore the importance of comprehensive preoperative assessment and procedural management in infection prevention. Overall, the present study underscores the importance of a multidimensional approach to infection prevention in gastrointestinal surgeries, integrating preoperative risk assessment, procedural optimization, and targeted interventions to enhance patient safety and outcomes.
